# Supplementary material for: Splice-Junction-Based Mapping of Alternative Isoforms in the Human Proteome
Source: Cell Rep. Author manuscript; Available in PMC 2020 Jan 15. (PMC6961840; doi:10.1016/j.celrep.2019.11.026)
Supplement: 2 [file NIHMS1546469-supplement-2.zip › DF1/PXD010154/Lung/Lung_37_MAF1_GDLPGEAINSQLTVETGDAHIIGR.pdf]

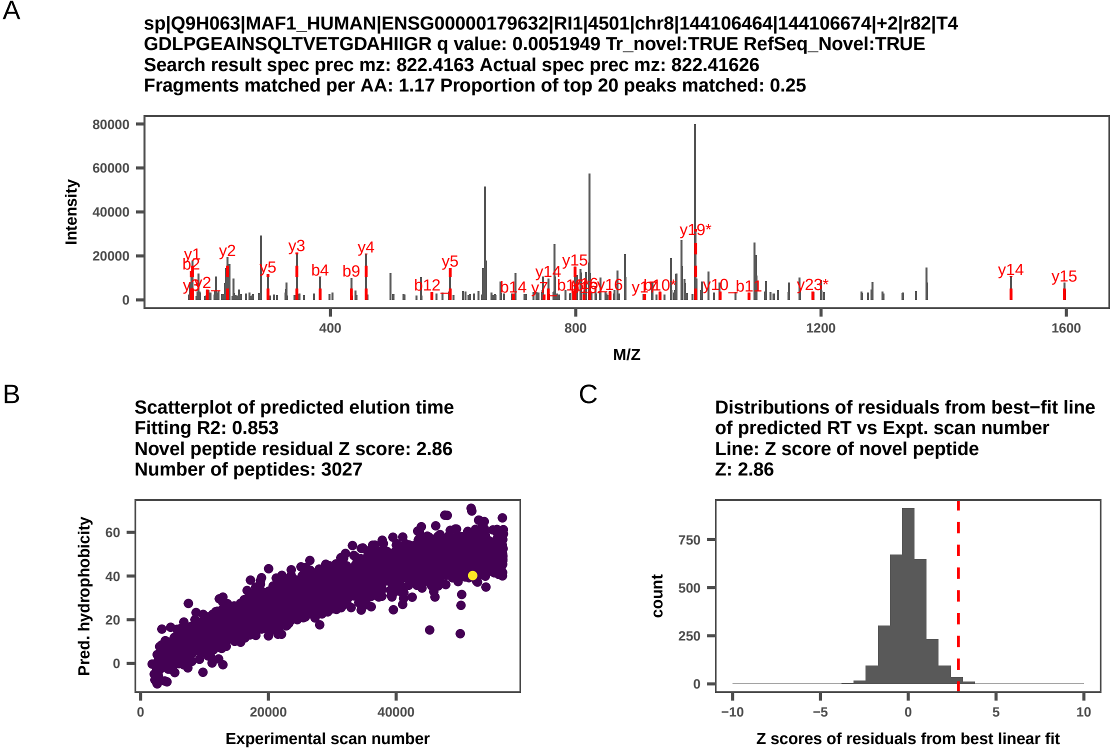
A
sp|Q9H063|MAF1_HUMAN|ENSG00000179632|RI1|4501|chr8|144106464|144106674|+2|r82|T4
GDLPGEAINSQLTVETGDAHIIGR q value: 0.0051949 Tr_novel:TRUE RefSeq_Novel:TRUE
Search result spec prec mz: 822.4163 Actual spec prec mz: 822.41626
Fragments matched per AA: 1.17 Proportion of top 20 peaks matched: 0.25
Intensity
80000
60000
40000
20000
0
y1
b2
y2
y3
y5
b4
b9
y4
y5
b12
b14
y14
y15
y19*
y10
b11
y23*
y14
y15
400
800
1200
1600
M/Z
B
Scatterplot of predicted elution time
Fitting R2: 0.853
Novel peptide residual Z score: 2.86
Number of peptides: 3027
Pred. hydrophobicity
60
40
20
0
0
20000
40000
Experimental scan number
C
Distributions of residuals from best−fit line
of predicted RT vs Expt. scan number
Line: Z score of novel peptide
Z: 2.86
count
750
500
250
0
−10
−5
0
5
10
Z scores of residuals from best linear fit
